# Function of translationally controlled tumor protein (TCTP) in *Eudrilus eugeniae* regeneration

**DOI:** 10.1371/journal.pone.0175319

**Published:** 2017-04-12

**Authors:** Elaiya Raja Subramanian, Nino Gopi Daisy, Dinesh Kumar Sudalaimani, Kalidas Ramamoorthy, Subburathinam Balakrishnan, Jackson Durairaj Selvan Christyraj, Vaithilingaraja Arumugaswami, Sudhakar Sivasubramaniam

**Affiliations:** 1 Department of Biotechnology, Manonmaniam Sundaranar University, Tirunelveli, Tamilnadu, India; 2 Viral Hepatitis and Gene/Cell Therapy Laboratory, CSMC Viral Vector Core, University of California, Los Angeles, United States of America; Jinling Institute of Technology, CHINA

## Abstract

TCTP (Translationally Controlled Tumour Protein) is a multifunctional protein that plays a role in the development, immune system, tumour reversion, and maintenance of stem cells. The mRNA of the *Tpt1* gene is over-expressed during liver regeneration. But, the function of the protein in regeneration is not known. To study the role of the protein in regeneration, the earthworm *Eudrilus eugeniae* was chosen. First, the full length cDNA of the *Tpt1* gene was sequenced. The size of the cDNA is 504 bp and the protein has 167 amino acids. The highest level of TCTP expression was documented in the worm after three days of regeneration. The protein was found to be expressed specifically in the epithelial layer of the skin. During regeneration, the protein expression was found to be the highest at the tip of blastema. The pharmacological suppression of TCTP using nutlin-3 and TCTP RNAi experiments resulted in the failure of the regeneration process. The suppression of TCTP caused the arrest of proliferation in posterior amputated worms. The severe cell death was documented in the amputated region of nutlin-3 injected worm. The silencing of TCTP has blocked the modification of clitellar segments. The experiments confirm that TCTP has major functions in the upstream signalling of cell proliferation in the early regeneration process in *E*. *eugeniae*.

## Introduction

The Translationally Controlled Tumour Protein (TCTP) is encoded by the gene *Tpt1* and is an evolutionarily conserved gene from yeast to human. Early reports suggested that TCTP is a tumour protein [1]. Later studies have revealed that TCTP has a vast number of functions in biological systems. The immune cells like T-lymphocytes [2], basophils and mast cells [3] have secreted the protein TCTP/HRP, which is associated with immunological responses of cells [2,3]. It has been reported that TCTP is a calcium binding protein [4] and plays a pivotal role in microtubule stabilization [5]. Furthermore, TCTP is associated with the cellular cytoskeleton to determine cell shape and migration [6,7]. TCTP acts as an anti-apoptotic protein [8,9]. The higher expression of TCTP suppresses the expression of tumour suppressor protein p53 [8] and VHL [10]. The pharmacological activation of p53 using nutlin-3, sertraline and thioridazine promotes TCTP degradation in cancer cells [11]. Nutlin-3 is a small molecule which inhibits the binding of p53-MDM2 and stabilizes endogenous p53 levels *in vitro* and *in vivo* [12,13]. The suppression of TCTP in tumour cells results in tumour reversion [14]. Interestingly, the protein TCTP is involved in the development and growth of both vertebrates and invertebrates [15,16]. The homozygous null mutant of TCTP shows lethality in mouse and *Drosophila* embryos. The report reveals that TCTP plays a major role in embryonic development [15,16]. In addition, higher expression of TCTP is noted in the early embryonic cleavage of Amphioxus but fails to be expressed in the later cleavage stage [17]. It is well known that embryogenesis and regeneration are dynamic processes which is regulated by stem cells [18,19] and signalling pathways [20]. Interestingly, Koziol et al., 2007 reported that TCTP regulates the stem cell factors Oct4 and Nanog in the *Xenopus* oocyte [21]. It also acts as an upstream molecule in several signalling pathways [15,22–24]. This ample amount of evidence confirms higher expression of TCTP in proliferation-rich tissues. However, the constant role of TCTP in regeneration process remain unknown.

Regeneration is a remarkable mechanistic process in organisms. The regeneration process in animals poses initial wound healing processes. Afterwards, the undifferentiated, newly formed stem cells/progenitor cells aggregate at the stump region. This is called the regeneration blastema. In a later stage, the cells undergo lineage-specific differentiation and re-form the lost parts. The adult stem cells [25–28] and several signalling pathways, such as Wnt [29], Src [30], Akt/PI3-k [31] and Notch [32], are highly involved in the regeneration process. The role of TCTP as a growth regulator in regeneration has not been clearly studied. There are very few reports that have spotted the expression of TCTP mRNA in regenerating tissues. It has been reported that the upregulation of TCTP mRNA is found in liver regeneration and liver cancer tissues [33]. The spatiotemporal expression of TCTP mRNAs was noted in the visceral regeneration of the sea cucumber [34].

The regeneration ability varies among the animals. In nature, the invertebrates secure the top position in the ability of regeneration.Compared to the invertebrates, the vertebrates have limited regeneration ability [35,36]. The higher fecundity rate, easy maintenance and wonderful regeneration ability of *Eudrilus eugeniae* made it a reliable model for the regeneration study. Our previous reports show that *E*. *eugeniae* regains its lost anterior and posterior parts through regeneration process [37,38].

In current studies, the cDNA of TCTP has been identified and sequenced. The protein sequence of TCTP in *E*. *eugeniae* has 80% homology with its human counterpart. Higher expression of TCTP was found in the early stage of the blastema. The administration of pharmacological inhibitors and specific siRNA against TCTP halts the regeneration process by disturbing the upstream cell differentiation in the clitellar segments.

## Materials and methods

### Worm maintenance

The culture stocks of *E*. *eugeniae* are rearing in the Department of Biotechnology, Manonmaniam Sundaranar University, Tirunelveli. The worms were maintained as per the protocol of published papers [37,39]. The worms were fed with an equal ratio of leaf litter, cow dung and were maintained in plastic container at appropriate moisture condition.

### Amputation of *E*. *eugeniae*

To study the posterior regeneration process, the mature worms were selected by the presence of a clitellum and were amputated at the posterior thirtieth segment using a sterile surgical blade. The amputated worms were maintained in appropriate conditions for respective analysis.

### RNA isolation and cDNA synthesis

The RNA isolation was performed as per the manufacturer’s protocol (Sigma). The tissue samples of *E*. *eugeniae* were dissected and washed with 1x ice cold PBS (prepared in DEPC water). The tissue was then ground with 10 mg/ml Trizol reagent (Cat.T9424; Sigma- Aldrich, India) using a glass homogenizer while on ice. After a five minute incubation period, the aqueous phase was separated and an equal amount of isoproponal was added for RNA preparation. The pellet was washed with 75% ethanol and dissolved in nuclease free water and then centrifuged. The first strand cDNA synthesis was performed using Transcriptor First Strand cDNA synthesis kit (version 6.0; Roche, Germany) as per manufacturer protocol (Roche).

### Identification and sequencing of TCTP

In order to amplify the gene TCTP, 20 ng of cDNA was taken and 30 PCR cycles were carried out in a SmartPCR-PRO Thermal cycler (SmartPCR-PRO; Cyberlab, USA) using the forward (5’ ATGATCATCTTCAAGGACG 3’) and reverse (oligo d (A)) primers (VBC biotech, Vienna, Austria) with denaturation at 94°C for 1 min, annealing at 59°C for 1 min, and polymerization at 72°C for 1 min. The PCR reaction mixtures were electrophoresed in 1% agarose gel, a 504 bp band was sliced out and the purified samples were used for sequencing.

### Multiple sequence alignment of TCTP from various animal sources using Align X program

The cDNA sequence of *E*. *eugeniae* TCTP was translated using the ExPASy translate tool and the TCTP sequences of different animals were retrieved from an NCBI source. The multiple sequence alignment was performed using Align X of Vector NTI package. The accession numbers for the TCTP of *Homo sapiens*, *Mus musculus*, *Danio rerio*, *Drosophila melanogaster*, and earthworm *Lumbricus rubellus* are AAQ01550.1, NP_033455.1, NP_937783.1, NP_650048.1, and CAA69350.1, respectively.

### Raising of *E*. *eugeniae* TCTP antibody

The synthetic peptide of *E*. *eugeniae* TCTP ^**60**^**ESTSKQGVDIVMNSRLVEF**^**78**^ was conjugated with a keyhole limpet hemocyanin (KLH) and immunized in rabbits. The anti-sera of the rabbit were used as an anti-TCTP antibody (Chromous biotech, India). The pre-immune serum was used for control experiments.

### SDS PAGE and Immunoblot analysis

SDS PAGE and Immunoblot analysis was perfomed using the protocol of previously published papers [40]. The respective tissues of *E*. *eugeniae* were obtained and gently washed with ice-cold 1X PBS. The individual tissues were ground with 2x sample buffer and 30 μg of tissue lysates were resolved in 12% SDS-PAGE. The proteins were transferred to the PVDF membrane (Cat. 1620177; Biorad, USA) and the western blot was conducted with the following primary antibodies: anti-TCTP antibody (Chromous biotech, India) and anti- β-actin antibody (Cat.A 2066; Sigma Aldrich, India), in the dilution of 1:1000 and 1:3000, respectively. The primary antibody incubation was carried out for 12 hours at 4°C. Followed by washing, the Goat anti-rabbit IgG–an alkaline phosphatase conjugate secondary antibody (Cat. A 9919; Sigma Aldrich)—was used to locate the primary antibody. The colorimetric substrate, BCIP/NBT (Cat. E116 ameresco, USA), was used as a developer solution. The developed membranes were documented by ChemiDoc XRS; Bio-Rad, USA and the intensity was estimated by image lab-2 analysis software (Bio-Rad, USA).

### Histology

The histological experiments were carried out to observe the cellular arrangements of *E*. *eugeniae* tissues in all the experiments. The respective tissue samples of *E*. *eugeniae* were sliced and fixed using 10% formaldehyde (Cat. MB059; Himedia, Mumbai, India) for 24 hours. To remove formaldehyde, the tissues were gently washed with distilled water and dehydration was carried out in 60%-100% of isopropanol (Cat. MB063; Himedia, Mumbai, India) for 1 hour each. After clearing with xylene, the tissues were incubated in paraffin wax (Cat. GRM1137; Himedia, Mumbai, India) for wax impregnation and the 6 μm sections were made using microtome (Cat. MT 1090A; weswax, India). Then, the wax was removed and the tissues were stained with Mayer’s hematoxylin (Cat.MHS1; Sigma-Aldrich, India) and eosin (Himedia, Mumbai, India). The samples were mounted with D.P.X (Himedia, Mumbai, India). The slides were observed under OLYMPUS BX53 upright 3 viewer microscope.

### Immunohistochemistry

The various experimental tissue sections of *E*. *eugeniae* were subjected to IHC by the protocol of published paper [40]. 6 μm sections were subjected to immunohistochemistry to observe the TCTP expression. After the paraffin was removed from the sections, they were treated with 10% H_2_O_2_ (Cat.1072090500; Merck, India) and 10% methanol (Cat. AS059; Himedia, India) in 1X PBS to block the endogenous peroxidise activity, followed by trypsin treatment (0.1% trypsin in 0.1% CaCl_2_ for 10 minutes). The non-specific blocking was carried out by keeping the tissue slides in 2% BSA for 1 hour at room temperature. The IHC was performed individually by primary antibodies of anti-TCTP antibody, anti-Phospho H3 [pSer^10^] antibody (Cat. H0412, Sigma-Aldrich, India) and mouse anti-BrdU antibody (Cat. B8434, Sigma-Aldrich, India) at a concentration of 1:100, 1:200, 1:100, respectively, in 2% BSA, overnight at 4°C. After the PBS wash had been rendered, sections were incubated with secondary antibody Goat anti-mouse IgG conjugated with horseradish peroxidise (Cat.031050, Sigma-Aldrich, India) at a dilution of 1:2000 in 1X PBST. Diaminobenzidine (Cat.E733; Amresco, USA) was used as a developer and sections were counter-stained with Mayer’s hematoxylin (Cat. MHS1, Sigma-Aldrich). The slides were mounted with DPX and documented under an Olympus BX53 microscope.

### Nutlin-3 injection

For nutlin-3 injection, forty matured worms were divided into four groups. Each group contains ten worms. The first two group of worms were injected with 5 μg/g concentration of nutlin-3 (Cat. N6287; Sigma-Aldrich, India) at the twenty-fourth segment. The stock solution of nutlin-3 was prepared in DMSO at the concentration of 0.1 mg/ml (Cat.TC185; Himedia, Mumbai, India). The working solution was obtained by diluting it with 1x PBS. The third group of worms were injected with 0.1% DMSO at the twenty-fourth segment. The rest of the fourth group of worms were used as a non-injected control. The first group of worms was maintained without amputation and the rest of the three groups were amputated at the posterior thirtieth segment and allowed for regeneration. The phenotypical changes of animals were photographed using a Canon digital camera. The worms were sacrificed on the fifth day post-amputation for further analysis.

### Designing of siRNA

The *E*. *eugeniae* TCTP mRNA sequence and the retrieved GFP mRNA sequence were used for designing the siRNA molecule using the BLOCK-iT™ RNAi Designer designing tool based on guidelines of Tuschl and colleagues. The designed TCTP siRNA and the GFP control siRNA were purchased from Eurofins Genomics, Germany.

TCTP siRNA

sense strand- 5'GGGAGUUGACAUCGUUAUGAA3';

antisense strand- 5' UUCAUAACGAUGUCAACUCCC3'

GFP siRNA

sense strand—5’UGGCUUUCUGUAGAGGACAUC3’;

anti sense strand– 5’GUTGTCCTCTUCUGUUUGCCU 3’.

### RNAi knock down of TCTP

As per the manufacturer protocol, the stock solution of siRNA was suspended in 1X siMAX buffer. Thirty worms were amputated at the thirtieth segment and the worms were divided into three groups. Each group has ten worms. Immediately after amputation, a 3 nM concentration of TCTP siRNA was injected at the twenty-fourth segment of each worm in the first group. The second set of worms was injected with 3 nM GFP control siRNA, as mentioned above. The third group of worms was maintained as a non injected control. Followed by the first injection, on third day, the 3 nM concentration of both TCTP and GFP siRNA were injected in respective groups. The phenotype of RNAi along with the control worms were photographed and sacrificed on the fifth day post-amputation for further analysis. The experiment was repeated thrice for further authentication of results.

### Tunnel assay

Tissues from worms (nutin-3 treated, TCTP RNAi), control tissues (0.1%DMSO injected and GFP siRNA), and all the four samples were individually processed for tunnel assay to find the apoptotic rate. The de-parrafinized tissue sections were subjected to endogenous peroxidise blocking (Freshly prepared 10% H2O2, 10% methanol in 1x PBS for thirty minutes). Then, tissue sections were treated with Equilibration Buffer at room temperature for ten minutes and the samples were incubated for one hour with the reaction mixture, which contains terminal deoxynucleotidyl transferase (Cat. EP0161; Thermo Fisher, USA) and BrdU (Sisco research Laboratories Pvt.Ltd, Mumbai, India), except four control tissue sections. The BrdU nick-end-labelled tunnel positive cells were detected by IHC with an anti-BrdU antibody as explained in the previous section.

### Mitotic index

Ten random images were captured at 100X and the mitotic cells were counted manually thrice in each images. The mitotic index was calculated by dividing the number of total phospho H3 positive cells by the total number of cells.

### Statistical analysis

The expression levels of proteins were documented by ChemiDoc XRS; Bio-Rad, USA and intensity was estimated by image lab-2 analysis software (Bio-Rad, USA). The expression level of TCTP proteins were normalized with expression values of β-actin then the test groups were normalized with control groups. The statistical analysis performed (mean, standard deviation and standard error) and values were plotted in graph using GraphPad Prism 6.0.

## Results

### Identification of earthworm *E*. *eugeniae* TCTP

**Fig 1:**
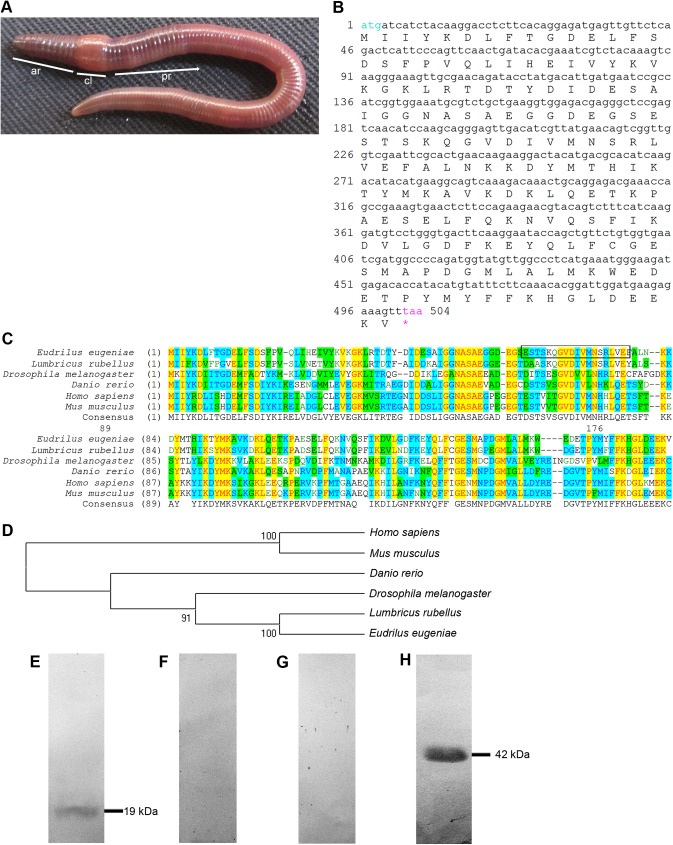
Morphology of *E*. *eugeniae* and identification of *E*. *eugeniae* TCTP. (A) The earthworm, *Eudrilus eugeniae*, ar-anterior region, cl-clitellum, pr- posterior region. (B) The full length cDNA sequence of *E*.*eugeniae* TCTP (*tpt1*) gene contain 504 bp. (C) Multiple sequence alignment of *E*.*eugeniae* TCTP protein sequence with its homologues of other animals using Align X of Vector NTI. It shows higher similarity of TCTP with the other animals including vertebrates. (D) Phylogenetic tree of *E*.*eugeniae* TCTP protein sequence with its homologues of other animals using Align X of Vector NTI. (E) Expression studies of *E*. *eugeniae* TCTP protein. The protein is 19 kDa in molecular weight. The 3^rd^ day regenerated tissue sample was analysed using immunoblot with anti-TCTP antibody. (F) Immunoblot with pre immune sera. It didn’t show any signals in PVDF membrane. (G) Immunoblot performed using anti- TCTP antibody with TCTP specific peptide. The peptide of TCTP which was used to raise the antibody, blocks the interact of the antibody with the TCTP molecules on the membrane. Each well was loaded with 40 μg of protein lyaste and the samples were resolved in 12% SDS-PAGE. (H) The expression of β-actin was used as a marker to confirm TCTP molecular weight.

The worm *E*. *eugeniae* is an annelid that has a segmented body (Fig 1A). The collar-like clitellum divides the worm into anterior and posterior region (Fig 1A). The *tpt1* (TCTP) gene on the cDNA was obtained from the available cDNA library by PCR reactions with TCTP specific forward (5’ ATGATCATCTTCAAGGACG 3’) and reverse (oligo d (A)) primer sets. The TCTP transcript was then sequenced (Fig 1B). The earthworm *tpt1*(TCTP) gene has totally 504 nucleotides (Fig 1B) and the mRNA encodes 167 amino acids. The protein sequences of its homologues in different animals were aligned **(Fig 1C)**. The multiple sequence alignment data (Fig 1C) and the phylogenetic tree analysis **(Fig 1D)** of TCTP displays higher homology of *E*. *eugeniae* TCTP sequence with that of other organisms. The TCTP sequence of *E*. *eugeniae* shows maximum homology with the TCTP of *Lumbricus rubellus* **(Fig 1D)**. To further study the expression of TCTP in the earthworm *E*. *eugeniae*, a peptide antibody was raised against the peptide region of the TCTP protein **(Fig 1E)**. In order to validate the presence of the anti-TCTP antibody, the immunoblot analysis was performed. The antibody detected a single band at 19 kDa in the immunoblot experiment carried out with lysate of the earthworm **(Fig 1E)** but the pre-immune serum didn’t show any bands in the experiment **(Fig 1F)**. To further confirm the specificity of TCTP antibody, the antibody was incubated with a TCTP peptide which was used to raise antibody. Then, it was used for an immunoblot experiment. The data shown in Fig 1. G clearly reveals that the peptide incubation with the antibody blocks the interaction of the antibody with TCTP molecule on the PVDF membrane **(Fig 1G)**. The expression of β-actin was used as a marker **(Fig 1H)**. The results confirm the specificity of the TCTP antibody.

### Expression of TCTP in *E*. *eugeniae* during regeneration

**Fig 2:**
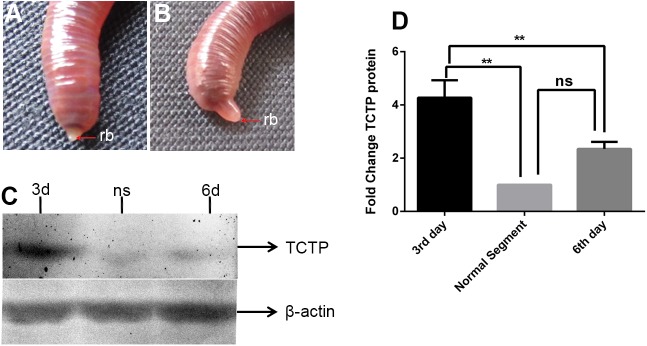
Higher expression of *E*.*eugeniae* TCTP during regeneration. (A) 3^rd^ day posterior regeneration, rb- regeneration blastema. (B) 6^th^ day posterior regeneration, rp-regenerated part. (C) Immunoblot of *E*.*eugeniae* TCTP using anti-TCTP antibody, lane 1, 2 & 3 shows the expression of TCTP in 3^rd^ regeneration blastema, normal segments and 6^th^ day regeneration tissues respectively. Each well was loaded with 40ug of protein lysate and the samples were resolved in 12% SDS-PAGE 3d-3^rd^ day, ns- normal segments, 6^th^ day. (D) On the third day of regeneration, the protein expression significantly higher and the expression significantly decreased at the 6^th^ day. Normalization TCTP expression was normalized with β-actin values. The values were given are mean ± SEM; **p <0.005

To examine the role of TCTP in *E*. *eugeniae* regeneration, ten worms were taken and amputated at the posterior thirtieth segment. In the amputation site, the blastema appeared on the third day **(Fig 2A)** and it increased in the size on the sixth day **(Fig 2B)**. The third day post amputation is a very early stage of the complex regeneration process in the worm. Our recent report reveals that the worm can complete the regeneration of functional anus on the sixth day [41]. Hence, the sixth day post-amputation is considered as mid-stage of the regeneration. In the later stage, the worm regains about eightieth segments in three months of time. The third and sixth day of blastema with the stump region and posterior segments of intact worms were dissected out and subjected to protein lysate preparation individually. All the protein samples were resolved in 12% SDS PAGE and subjected to immunoblot with an anti-TCTP antibody and anti-β-actin antibody individually. The experimental result shown in Fig 2C clearly reveals that the β-actin expression is relatively similar in all three samples **(Fig 2C)**. The segments of intact worm expressed the least amount of protein among the three samples but the third day regenerating worm expressed the maximum amount of TCTP. In contrast, the protein expression declined in the sixth day **(Fig 2C)**. To further corroborate the results, the experiment was repeated multiple times. The statistical analysis was performed and depicted in Fig 2D. The results reveal that, while comparing the protein expression in the intact worm segments, the third day regenerated part shows higher expression of TCTP, the result is considerably significant (*P* <0.005). On the sixth day, the protein expression declines significantly in comparison to the expression on the third day (*P* <0.005). The data clearly shows that early blastema and adjacent segments express the TCTP protein at maximal amount and in the later days, the protein expression declines sharply.

### Spatiotemporal expression of TCTP during *E*. *eugeniae* regeneration

**Fig 3:**
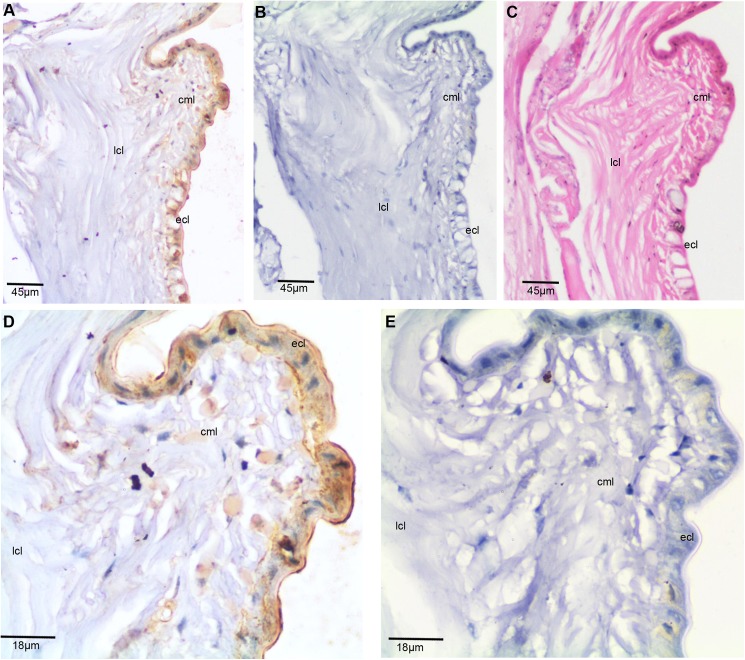
Expression of *E*. *eugeniae* TCTP in intact posterior segments of *E*. *eugeniae*. (A) Immunohistochemistry with anti-TCTP antibody using the intact posterior segments of *E*. *eugeniae*. It shows mere expression of TCTP at the epithelial cell region (B) The pre-immune serum treated sections used as a control, it didn’t show the signals. (C) The consecutive section of IHC sample was stained with eiosin and haemotoxylin. (D) 40X Magnified image of panel **A** shows the expression of TCTP protein ECL layer. (E) 40X magnified image of Panel B, show no positive signals.

The spatiotemporal expression of TCTP has been documented in several biological systems. The localization of TCTP has been found in both cytoplasm and nucleus of the cells [5,42–44]. TCTP is also secreted by many different cells in a vertebrate immune system [2,3,45]. In order to state the spatiotemporal expression of TCTP in the worm, immunohistochemistry (IHC) was performed on segments of the control, early (third day), and later (sixth day) blastemal tissues. Initially, the thirtieth-thirty-fifth posterior segment of the intact worm paraffin embedded tissue was sectioned. Three consecutive 6 μm sections were obtained. One of them was subjected to IHC with the anti-TCTP antibody **(Fig 3A).** With the second section, IHC was also carried out using the pre-immune serum for control purposes **(Fig 3B)**. In order to understand the tissue pattern, the last section was subjected to histological analysis **(Fig 3C)**. The skin of *E*. *eugeniae* is made with three different layers: ECL, CML, and LCL [37,38] and it is shown in **Fig 3A–3C**. The higher magnified image of panel **Fig 3A** shows most of cells in the outer epithelial cell layer expresses the protein (**Fig 3D)**. A few cells in the longitudinal layer also express the protein (**Fig 3D)**. The TCTP localization is found in the cytoplasm and the extra-cellular matrix region, but not in the nucleus. There are no signals in the tissue section processed with the pre immune serum (**Fig 3E)**. The data confirms the specificity of anti-TCTP antibody in the IHC experiment.

### TCTP expression in early regeneration

**Fig 4:**
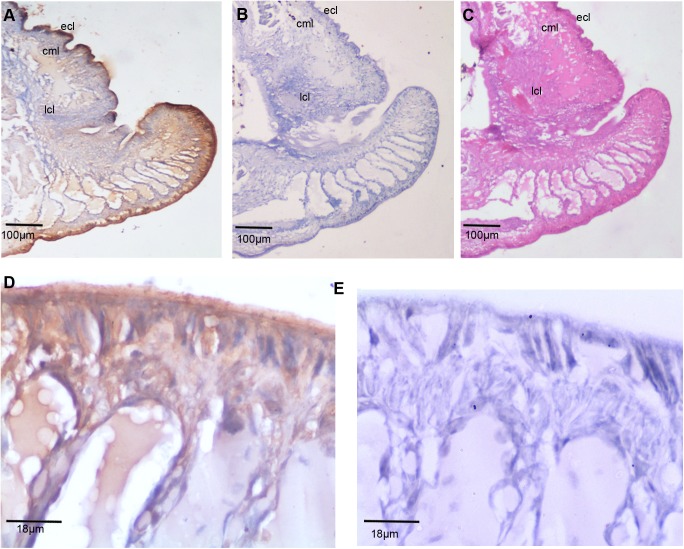
Expression of *E*. *eugeniae* TCTP in 3^rd^ day regeneration blastemal. (A) Immunohistochemistry with anti-TCTP antibody using the 3^rd^ day regeneration blastema. It shows abundant expression of TCTP at the blastemal region (B) The pre immune serum treated sections used as a control, it didn’t show the signals. (C) The consecutive section of IHC sample was stained with eiosin and haemotoxylin. (D) 40X Magnified image of panel **A** shows the rich expression of TCTP protein in the blastema. The tip of the blastema shows maximal the expression of TCTP. (E) 40X magnified image of Panel B, show no positive signals.

In order to study the expression of TCTP in early regeneration, five worms were amputated at the thirtieth segment. On the third day post amputation, the blastemal tissue, along with the two adjacent segments, were dissected and fixed with formalin fixative. The paraffin embedded thin sections were subjected to IHC with the anti-TCTP antibody **(Fig 4A)**. The consecutive sections were used for the control experiments as follows. One of the tissue sections was used for IHC with the pre-immune serum. There was no signal detected in control tissues **(Fig 4B and 4E)**. **Fig 4C** shows the tissue pattern in the blastema, which clearly illustrates that the ventral side tissue of the blastema is longer than the dorsal side. The data show the followings: 1. the cells in the terminal end of the blastema express the highest amount of TCTP **(Fig 4D)**; 2. Most of the cells in the outer most epidermal layer of *E*.*eugeniae* skin express the protein **(Fig 4D)**; 3. The fraction of cells of septum and skin longitudinal skin layer also express the protein and the level of TCTP expression is gradually reduced in the cells from the tip of blastemal tissue to the stump region **(Fig 4A and 4D)**.

### TCTP expression in the later stage of regeneration

**Fig 5:**
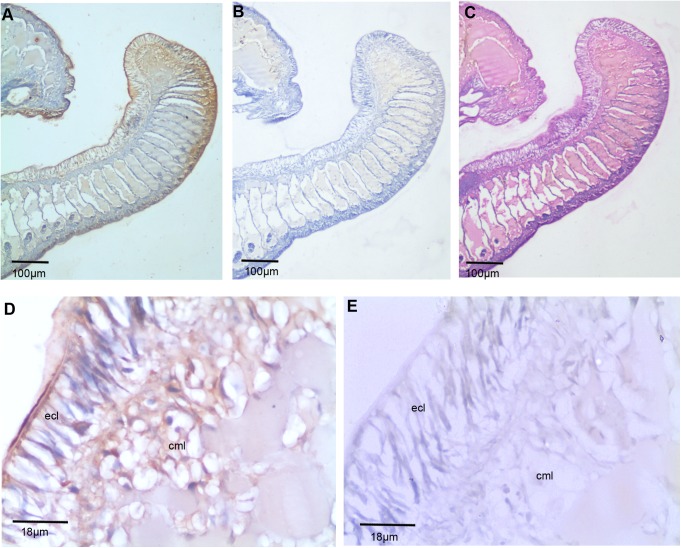
Expression and localization of *E*. *eugeniae* TCTP in 6^th^ day regeneration tissue. (A) Immunohistochemistry with anti-TCTP antibody using the 6^th^ day regeneration blastema. It shows expression of TCTP at the tip of the regenerated part (B) The pre immune serum treated sections used as a control, it didn’t show the signals. (C) The consecutive section of IHC sample was stained with eiosin and haemotoxylin. (D) 40X Magnified image of panel **A** shows the rich expression of TCTP protein in the regenerated part. The tip of the regenerated part shows maximal expression of TCTP. (E) 40X magnified image of Panel B, show no positive signals.

The expression of TCTP protein was examined on the sixth day of posterior regenerating *E*. *eugeniae*. The worms were amputated, as said in previous section, and on the sixth day, the regenerating part along with the two adjacent segments were subjected to IHC with anti-TCTP antibody **(Fig 5A)**. The pre-immune serum treated control **(Fig 5B and 5E)** and histology **(Fig 5C**) have been performed in consecutive tissue sections, respectively. The positive signal was not there in control experimental tissues **(Fig 5B and 5E)**. The histological image of the sixth day regenerated part clearly shows dorsally opened anus and it consists of dorsal and ventral regions **(Fig 5C)**. The IHC experimental results clearly show that the expression of TCTP found at the growing tip of both dorsal and ventral region of regenerating part **(Fig 5A)**. The expression was also noted in the intestinal lumen epithelial cells of growing tip **(Fig 5A)**. Alike to the expression of TCTP in early regeneration, the later (6^th^ day) regenerating part also has the intense signals in outer cell layers **(Fig 5D)**, but the expression level has been reduced significantly.

### Influence of nutlin-3 in TCTP expression and *E*. *eugeniae* regeneration

**Fig 6:**
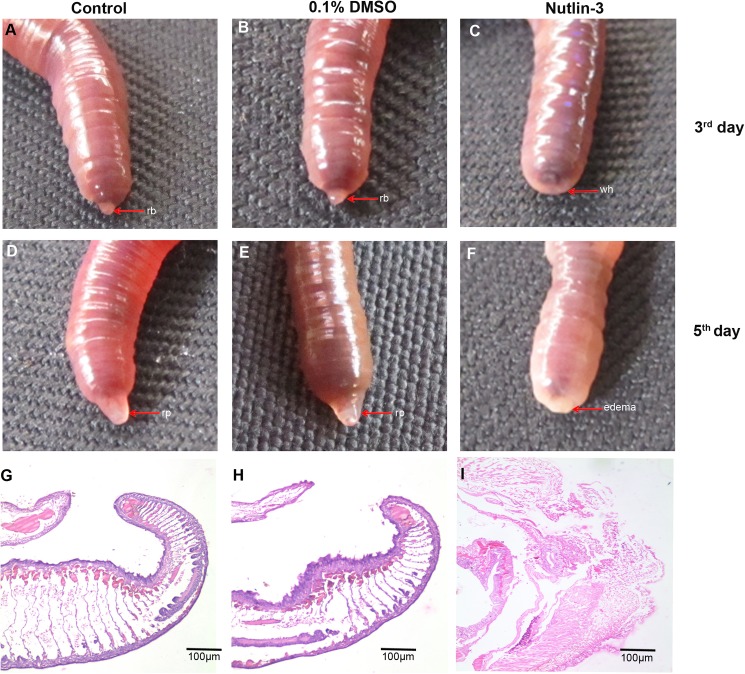
Regeneration of nutil-3 injected worm. **(A)** Non injected control animals, it formed the regeneration blastema on the 3^rd^ day. **(B)** 0.1% of DMSO injected worm, it formed the regeneration blastema on the 3^rd^ day **(C)** The worm injected with 5 mg/Kg concentration of nutlin-3 was amputated and maintain for regeneration. The worm on 3^rd^ day shows no regeneration blastema. **(D)** Control 0.1% of DMSO injected worm formed the regeneration blastema. **(E)** Non injected control animal formed the blastema as expected. **(F)** The nutlin-3 injected worm on 5^th^ day fails to form blastema even in fifth day. **(G)** Histological image (4X) of non injected worm tissue, it shows well developed regenerated part. **(H)** Histological image (4X) of 5^th^ day control 0.1% DMSO injected worm tissue, it shows well developed regenerated part. **(I)** Histological image (4X) of 5^th^ day nutlin-3 treated worm tissue, the cells are loosely packed at the amputated region.

The collective results of western blot and IHC suggested that TCTP is highly expressed in the regeneration tissues of *E*. *eugeniae*. The findings suggest that the protein may have an important function in the regeneration process and, previously, there was a report regarding the expression of the protein during regeneration of rat liver [33] and the visceral regeneration of sea cucumber [34]. For exploration of the protein function more, further experiments were executed using a pharmacological inhibitor of TCTP protein. Nutlin-3 is a selective pharmacological inhibitor of TCTP protein which can stabilise the tumour suppressor protein, p53 [11]. Several reports revealed that TCTP and p53 have the antogonestic expression pattern [8,11,46]. Often, the higher expression of p53 readily downregulates the expression of TCTP [11]. Hence, the nutlin-3 was injected to verify the prevalent role of TCTP in posterior amputated *E*. *eugeniae*. Forty worms were selected and divided into four groups. The first two groups were injected with 5 μg/g concentration of nutlin-3, which dissolved in DMSO at the posterior twenty-fourth segment. The third, control group was injected with 0.1% of DMSO and the fourth group was used as the non-injected control. After two hours, all the injected worms were amputated at the posterior thirtieth segment except the first group and maintained separately for regeneration analysis. Injection of nutlin-3 at 5 μg/g concentration didn’t cause any morphological impacts in the first group worms **(S1 Fig.)**. It is well known that the regeneration process of *E*. *eugeniae* started at the third day post amputation [37,38]. Similarly, blastema appeared at the stump region of all the control and non injected control worms **(Fig 6A and 6B)**. Interestingly, the blastema failed to appear in all the nutlin-3 injected worms, but the worm closed the wound at the amputation site **(Fig 6C)**. The worms were observed on the fifth day and it was found that nutlin-3 injected worms failed to regenerate the lost posterior part **(Fig 6F)**. It was also noted that the nutlin-3 injected worms were weak and morphologically abnormal **(Fig 6F)**. The worm body shrank and lost the intact body colour and the lateral sides of worm was changed into pale yellow colour **(Fig 6F)**. Moreover, the edema-like appearance was noted at the amputated region of nutlin-3 injected worms **(Fig 6F)** and nutlin-3 caused lethality in ±1 worm. Hence, the experiment was stopped on fifth day. However, the control and non-injected control animals regenerated the amputated parts successfully **(Fig 6D and 6E)**. The experimental results confirm that nutlin-3 inhibits the regeneration process of *E*. *eugeniae*. To examine the cellular pattern of nutlin-3 injected worms, histological experiments were carried out in nutlin-3, DMSO injected and the non-injected control worm tissues. The experimental result clearly shows that the nutlin-3 injected worm’s tissue of amputation site was structurally uneven and the cells were loosely packed at the site of amputation **(Fig 6I)**. In contrast, the DMSO injected and control worm regenerated the lost segments **(Fig 6G and 6H)**. Nutlin-3 heavily resulted abnormality in the cellular pattern of the amputation region of *E*. *eugeniae* **(Fig 6I)**.

**Fig 7:**
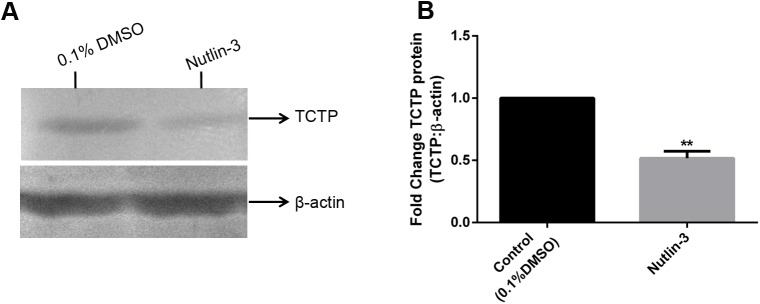
Influence of of nutlin-3 in TCTP expression on regeneration. **(A)** Expression of TCTP protein in the worm injected with 0.1% DMSO (Lane 1), Expression of TCTP protein in the worm injected with 5 μg/g nutlin-3 (Lane 2). Expression of β-actin in both and 0.1% DMSO and nutlin-3 injected worm (Lane 1, Lane 2, respectively). Each well was loaded with 40 μg of protein lysate and the samples were resolved in 12% SDS-PAGE. **(B)** The expression of TCTP significantly reduced in nutlin-3 treated injected sample. Normalization TCTP expression was normalized with β-actin values and the test groups were normalized with control group. The values were given are mean ± SEM; **p <0.005

To understand the expression level of TCTP in nutlin-3 injected worms, the tissue samples of worm injected with nutlin-3 and DMSO were subjected to immunoblot analysis with anti-TCTP antibody **(Fig 7A)**. The expression of β-actin protein was used as a control **(Fig 7A)**. Obviously, the experimental results clearly shows that the nutlin-3 injection drastically decreased the expression of TCTP **(Fig 7A)**. In contrast, the expression of β-actin was relatively similar in both the control and nutlin-3 injected worms **(Fig 7A)**. For quantification of the differences in the protein expression, the expression level of TCTP was normalised with β-actin and it was depicted in graph, which clearly shows the significant reduction of TCTP expression in nutlin-3 injected worms (*P <0*.*005*) **(Fig 7B)**. It is confirmed that nutlin-3 suppresses the TCTP expression in the worm as observed in the vertebrate [11] and it severely causes the impairment on the *E*. *eugeniae* regeneration.

### RNAi of TCTP in *E*. *eugeniae* regeneration

**Fig 8:**
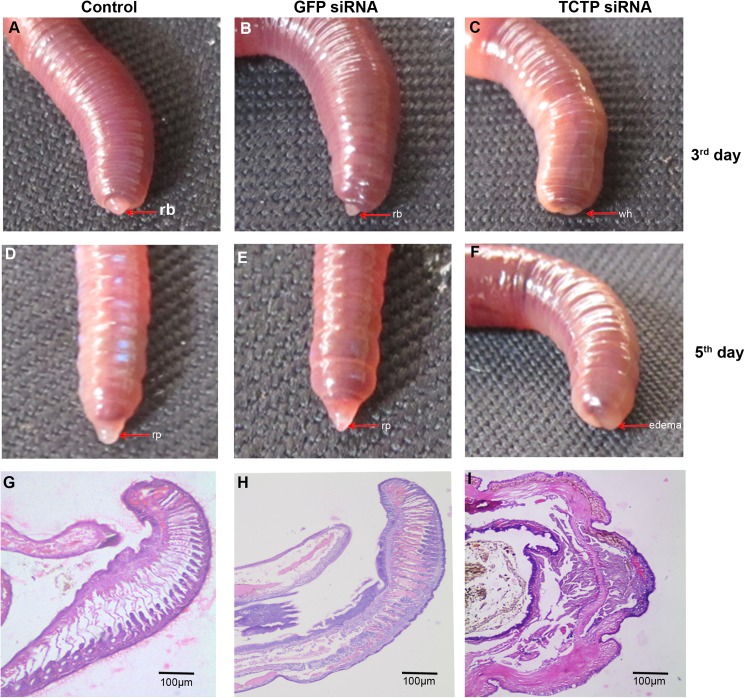
Effect of TCTP RNAi in the regeneration. **(A)** Non injected control worm formed the regeneration blastema on the 3^rd^ day. **(B)** The 6 nM GFP siRNA administrated worms were formed the regeneration blastema on the 3^rd^ day. **(C)** The 6 nM TCTP siRNA administrated worms shows no regeneration blastema on the 3^rd^ day. **(D)** The non-injected control animals reform the lost segments on the 5^th^ day. Similarly, **(E)** The 6 nM GFP control siRNA injected worm normally continued the regeneration process. **(F)** The 6 nM concentration of TCTP siRNA injected worm on the 5^th^ day, shows TCTP siRNA suppress the regeneration process. **(G)** Histological image (4X) of non injected worm tissue, it shows well developed regenerated part. **(H)** Histological image (4X) of 5^th^ day GFP siRNA injected worm developed regeneration blastema normally as expected. **(I)** Histological image (4X) of 5^th^ day TCTP siRNA injected worm tissue. It failed to form the regeneration blastema.

For further verification of the data obtained in experiments carried out with the administration of the nutlin-3 in the worm, the TCTP specific siRNA was synthesised for the target inhibition of endogenous TCTP mRNA during the regeneration. Fifteen worms were selected and amputated, as mentioned in the method section, and divided into three groups. Subsequently, the synthesised TCTP siRNA (3 nM) was administered at the twenty-fourth segment of the first group (as shown in method section). The second injection of siRNA was performed on the third day post-amputation. Similarly, GFP-specific siRNA was injected into the worms for the control purpose. The non-injected third group worms were used as a negative control. The regeneration blastema was appeard on 3^rd^ of amputation in GFP siRNA injected and non injected control worms **(Fig 8A and 8B)**. Interestingly, the RNAi of TCTP heavily affected the regeneration process of *E*. *eugeniae*. The TCTP siRNA injected worms phenotypically differs from the control and non-injected worms **(Fig 8A–8C)**. The worms injected with TCTP siRNA failed to generate the blastema on the third day **(Fig 8C)** and the worms received the second dose of TCTP siRNA on the third day. The TCTP siRNA injected worms didn’t generate the blastema on the fifth day, also **(Fig 8F)**. One of the TCTP siRNA injected worms was found dead on the fifth day. Hence, the administration of siRNA was stopped on the fifth day. The GFP siRNA and noninjected worms form apparently similar size of regenerated part **(Fig 8E and 8F)**. The series of data shown in **Fig 8** clearly confirms that TCTP protein is important for the formation of blastema and regeneration.

For further understanding of cellular pattern of worms injected with TCTP siRNA, GFP siRNA and non injected worms respectively were subjected for histological analysis. The data are shown in **Fig 8G–8I**, respectively. The worm injected with GFP siRNA and non-injected control worm generated the blastema **(Fig 8G and 8H)**. In contrast, the TCTP RNAi injected worm closed the wound at the amputation site by the outer epithelial cell layer but it failed to form the blastema **(Fig 8I)**.

**Fig 9:**
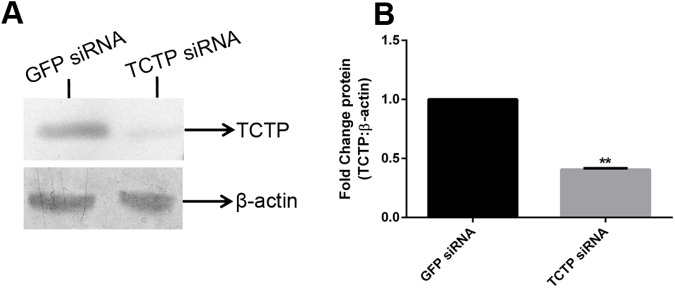
Knowdown effect and expression level of TCTP in TCTP RANi. (A) Expression of GFP control siRNA injected worm (Lane 1) Expression of TCTP protein upon TCTP siRNA administration (Lane 2). Expression of β-actin in GFP siRNA injected worm (Lane 1), and TCTP siRNA injected worm (Lane 2). Each well was loaded with 40 μg of protein lyaste and the samples were resolved in 12% SDS-PAGE. (B) The expression of TCTP significantly reduced in TCTP siRNA injected sample. Normalization TCTP expression was normalized with β-actin values and the test groups were normalized with control group. The values were given are mean ± SEM; **p <0.005

In order to assess the knockdown level of TCTP, immunoblot was performed and found that TCTP siRNA administration successfully silenced the expression of the protein on the fifth day post amputation in the worms **(Fig 9A)**. In contrast, the GFP specific siRNA molecule didn’t affect the expression of TCTP protein. Expression of β-actin protein was used to confirm the specific silencing of TCTP protein **(Fig 9A)**. To further authenticate the result, the results were quantified and graphed **(Fig 9B)**. The expression of TCTP was normalised with the expression of β-actin and control groups were normalized with test group the significant reduction of TCTP has been observed in TCTP RNAi worm (**Fig 9B)**. The RNAi experimental data clearly confirms that TCTP play a pivotal role in *E*. *eugeniae* regeneration and suppression of TCTP cause the regeneration arrest. In order to authenticate the results, the same RNAi experiments were repeated with the same outcome.

**Fig 10:**
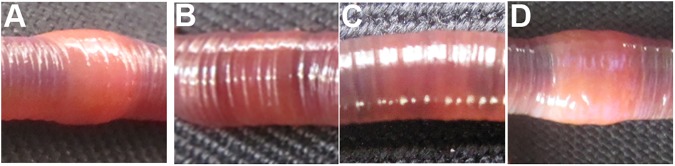
Phenotypical abnormality of clitellum during TCTP RNAi. (A) Clitellum of intact animal shows puffy appearance. (B) Clitellum of 5^th^ day regenerated worm, it lost the puffy nature. (C) Clitellum of the 5^th^ day regenerated GFP siRNA injected worm, it lost the puffy nature. (D) Clitellum of TCTP siRNA injected worm, it retains the puffy nature of the control worm.

The clitellum is a thick, collar-like, cylindrical part of *E*. *eugeniae* **(Fig 10A)**. The clitellar puffiness disappeared on the fifth day post amputation **(Fig 10B)**. A similar change (the loss of puffiness) of the clitellar segments has also been documented in GFP siRNA injected worms (**Fig 10C)**. Interestingly, the morphological changes in the clitellar segments were not observed in the worms injected with TCTP siRNA (**Fig 10D)**. The data suggests that, during regeneration, the clitellum undergoes obvious morphological change which requires the TCTP protein.

**Fig 11:**
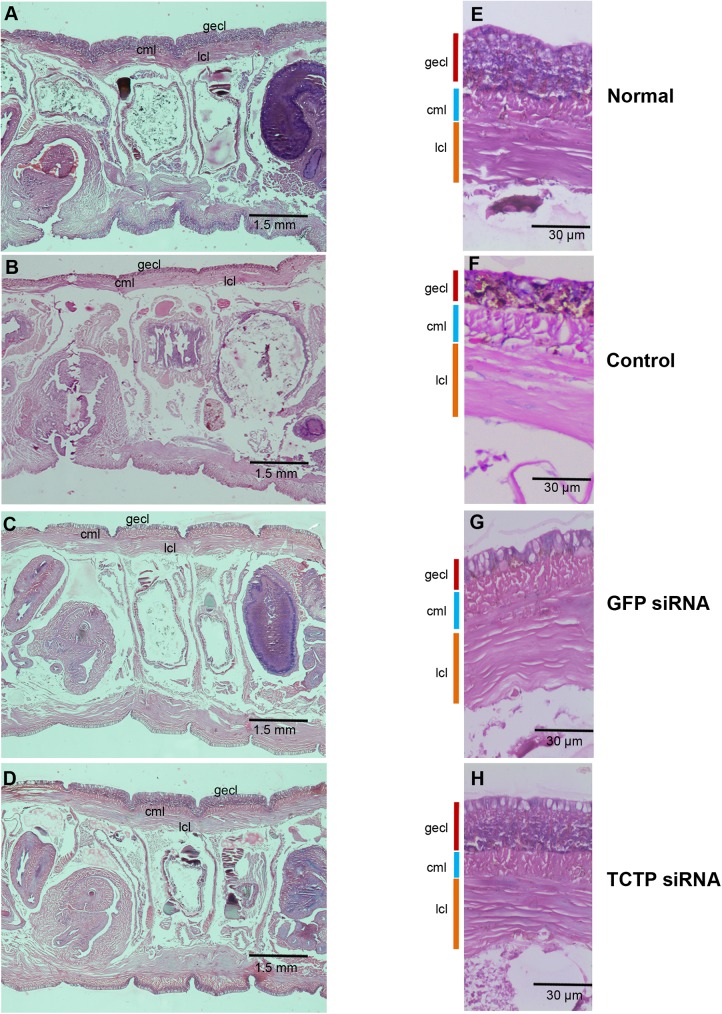
Cellular abnormality of clitellum during TCTP RNAi. **(**A and E) 3X and 10X magnified histological image of intact **c**litellum shows thick glandular epithelial cell layer (gecl) (B and F) 3X and 10X magnified histological image of the 5^th^ day regenerating worm, it shows drastic reduction of glandular epithelial cell layer in the clitellar segments. (C and G) 3X and 10X magnified histological image of the 5^th^ day regenerating GFP siRNA injected worm, it shows drastic reduction of glandular epithelial cell layer in the clitellar segments. (D and H) 3X and 10X magnified histological image of the 5^th^ day TCTP siRNA injected worm, it retains the structure of control worm’s clitellar glandular epithelial cell layer.

The normal, fifth day regenerating, TCTP and GFP siRNA injected worms clitellum were subjected to histological analysis (**Fig 11A–11D)** to understand the changes in cellular pattern. As described earlier, the skin of *E*. *eugeniae* is made with three different layers: the outer epithelial cell layer (ECL), the circular muscle layer (CML) and the inner longitudinal cell layer (LCL) (**Fig 11A)**. The ECL skin of clitellar segments is much thicker in the clitellar segments (**Fig 11A and 11E)** and the cells act as secreting cells, hence, the ECL of clitellum is called as glandular epithelial cell layer (GECL) [47]. In the course of regeneration, the worms lost their GECL and it changed into normal segmental epithelial cell like structure (**Fig 11B and 11F)**. Obviously, the same was observed in the GFP siRNA injected worms (**Fig 11C and 11G)**. Contrastingly, no change of GECL occurred in the TCTP RNAi worms (**Fig 11D and 11H)**. The size of the GECL of TCTP RNAi worms were apparently similar to the control worm GECL (**Fig 11A, 11D, 11E and 11H)**. The observation confirms that TCTP protein has functional link in the phenotypical changes of GECL during regeneration. The molecular role of the TCTP protein during the regeneration is unknown.

### Mitotic index

**Fig 12:**
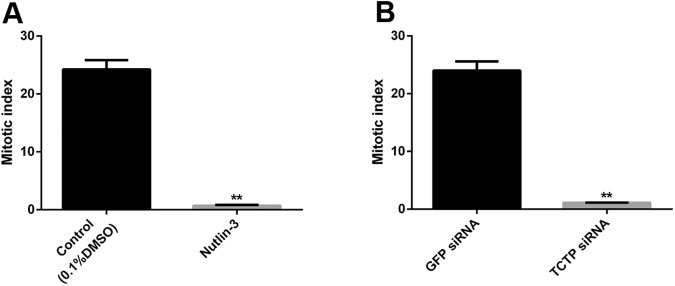
Mitotic index. (A) Mitotic index of the nutlin3 treated and control 0.1% of DMSO injected worms. It shows significantly low mitotic activity at the nutlin3 treated sample. (B) Mitotic index of the TCTP siRNA injected and control GFP siRNA injected worm, it shows significantly low mitotic activity at the TCTP siRNA sample. The values were given are mean ± SEM; **p <0.005

It is well known that mitosis is more prevalent in actively growing parts of organisms. Regeneration is the active tissue reclamation process which is made with higher cell turnover such as cell proliferation and differentiation. The previous reports suggested that TCTP plays a profound role in mitosis [5,48]. To examine the mitotic effect upon TCTP suppression in the regenerating worm, the mitotic index assay was conducted using anti-phosho serine^10^ histone H3 antibody. In order to distinguish the mitotic activity of control and TCTP suppressed worms (nutlin-3 injected, TCTP RNAi), the mitotic cells were counted in those tissue sections and plotted in a graph (**Fig 12A and 12B)**. The experimental result confirms the presence of a few phospho serine^10^ histone H3 positive cells in the nutlin-3 injected worm (**Fig 12A)**. In addition, TCTP RNAi also blocked mitosis in the tissues of regenerating part but more mitotic cells were documented in the regenerating parts of worm injected with GFP specific siRNA (**Fig 12B)**. The data confirmed that TCTP has major role in the mitosis during regeneration process, as well.

### Tunnel assay

**Fig 13:**
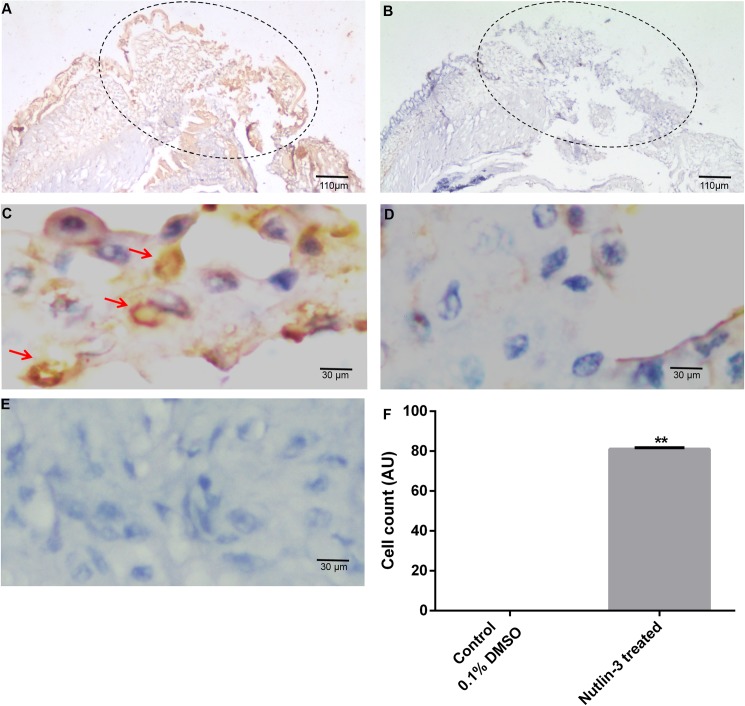
Tunnel assay. **(A)** 4X image of tissue section of worm administrated with nutlin3 and subsequently subjected to tunnel experimental. The brown cells are apoptotic cells (**B)** 4X image of tissue section of worm administrated with nutlin3 and subsequently subjected to tunnel experimental without the enzyme terminal transferase. The brown cells apoptotic cells are not observed. **(C)** 100X Magnified image of panel A, the nuclear signals noted in tunnel positive cells. **(D)** 100X Magnified image of panel B. **(E)** 100X image of 0.1% DMSO injected worm tissue shows no tunnel positive cells **(F)** The graph shows the significant difference in the apoptotic cells in the nutilin-3 and DMSO injected worms. The values were given are mean ± SEM; **p <0.005

The vast number of reports suggested that TCTP is a master anti-apoptotic protein which is negatively regulating the programmed cell death in both *in vitro* [49] and *in vivo* [16] systems. To verify the programmed cell death, the worms, which were individually injected with the nutin-3 and TCTP RNAi, were subjected to the tunnel assay. The thin sections of nutlin-3 injected tissues were incubated with terminal deoxynucleotidyl transferase mediated BrdU nick end labelling method (**Fig 13A)**. The control experiment was performed without the addition of terminal deoxynucleotidyl transferase enzyme in the tunnel reaction mixer (**Fig 13B)**. In order to locate the nucleus of the cells, all the experimental tissues were counter-stained with haematoxylin. The experimental results showed the presence of a higher number of bright brown-colour tunnel positive cells in the amputated region of nutlin-3 injected samples, but not in the control tissues (**Fig 13A and 13B)**. To further verify the tunnel positive signals, the sections were visualized under higher magnification and the nuclear signal of tunnel positive cells were documented (**Fig 13C)**. Obviously, there were no tunnel positive cells found in the control experiment (**Fig 13D)**. In addition, the tunnel experiment was performed in the 0.1% DMSO treated tissues and found the absence of apoptotic cells (data not shown here). The tunnel positives cells were compared with 0.1% DMSO treated tissues and plotted in graph (**Fig 13G)**. In addition, the TCTP RNAi and its control tissues also failed to show tunnel positive cells **(S2 Fig)**.

## Discussion

The evolutionarily conserved nature **(Fig 1C and 1D)** of the multi functional protein TCTP confirms its importance in living system. It has a prevalent role in growth and development of both plants [48,50] and animals [15–17]. It is interesting to note that the TCTP protein sequence of *E*. *eugeniae* and *Lubricus rubulus* is not identical **(Fig 1C)** and they have 95% homology. The same difference in homology has been observed between human and mouse (**Fig 1C**). The observation reveals that the significant differences also found among earthworm species.

The higher expression of TCTP in the third day of regeneration **(Fig 2A)** suggests that the protein has a role in the early regeneration process. Similarly, the higher expression of TCTP mRNA has been found during an early stage of liver regeneration in rats [33]. The expression level observed in the immunoblot analysis was further confirmed in the experiment of IHC which was carried out with the third and sixth day regenerating earthworm (**Figs 4A and 5A)**. Diminishing of the protein on the sixth day further confirms the specific expression of the protein during early regeneration. The maximal expression of the protein in the tip of blastema (**Figs 4A and 5A)** supports the finding of Munch et al., (2013). In the zebra fish fin regeneration, the cells in the tip region of the blastema expresses the signalling molecules [32]. It has been reported that the TCTP regulated the signalling pathway of Wnt [23], Src [51], Akt/PI3-k [22], and p53 [8]. In addition, Chen *et*. *al* observed the higher expression of TCTP mRNA at the early cleavage of amphioxus embryo and its expression decreased drastically in later stage of development [17]. The collective reports and present set of experiments confirm that TCTP play a major role in both early development and in early regeneration. This is the first report which elucidated the TCTP expression in regeneration at the protein level.

The observation of thousands of cells in the IHC experiments concludes the expression of the protein in the cytoplasm, extra cellular matrix and outer layer of *E*. *eugeniae* skin (**Figs 4D and 5D)**. Similarly, vast number of reports show the cytoplasmic localization and various functions of TCTP in different systems. The cells of immune system secrete the protein [3,45] and the localization has been found predominantly in the cytoplasm [52–54]. TCTP is secreted through exosomes by tumor-suppressor-activated pathway-6 (TSAP6) via a nonclassical pathway and the association of TCTP and TSAP6 in cytoplasm has been documented [54]. The protein is also present in the prostatic fluids [55] and the prostate epithelial cells has the protein in the cytoplasm during interphase [55] and it is associated with tubulin during mitosis [5].

It is known that the Na,K pump occurs in the cell membrane [56]. TCTP is involved in the regulation of Na,K-Atpase activity via Src [51] and it activates the signaling pathways of Akt/PI-3 Kinase [22]. The localization of the protein in the cytoplasm is well confirmed and there is no contrary report. On the other hand, TCTP stabilizes the anti-apoptotic protein by the degradation of tumour suppressors p53 and VHL in the cytoplasm of cells [8–11]. In current observation, the expression of the protein in the outermost skin layer and localization in the extracellular matrix region and cytoplasm **(Figs 4A and 5A)** suggests that it might have multiple roles during *E*. *eugeniae* regeneration.

On the contrary, some of the reports show that the protein expression has also been found in the nucleus of the cells [44,57]. TCTP binds with the promoter of Oct4 in the transcriptional activation of stem cell factors Oct4 and Nanog [21] and it also activates the transcriptional activity of Wnt signaling molecule, TCF4 [23]. The stem cell factors and Wnt signaling molecules are important for development and regeneration [20,58]. In the present study, the nuclear localization of TCTP is not clearly documented in *E*. *eugeniae* tissues. The higher expression of p53 downregulates both stems cell factors (Oct4, Nanog) and beta catenine is a key Wnt signaling molecule [59,60]. The injection of nutlin-3 downregulated the stem cell factors (Oct4, Nanog) by the activation of p53 and the higher experession of p53 cause proteosomal degradation of Wnt beta-catenin [60]. According to this report, it was assumed that TCTP might maintain the stem cell factors and Wnt signaling by the way of p53 suppression in cytosol of *E*. *eugeniae* regeneration.

It is interesting to observe the nutlin-3 injection down regulated the expression of TCTP in worm also (**Fig 7C and 7F)**. The observation confirms that the p53 pathway is conserved from vertebrate to the annelids. The regeneration arrest and death of worms on the fifth of regeneration caused by the administration of nutlin-3 and the silencing of TCTP with specific siRNA successfully confirms the function of TCTP in regeneration. The previous report found TCTP to be an important molecule for early development. The homozygous mutant of TCTP readily caused the lethality in mice embryos [16] and TCTP null mutant in *Drosophila* causes lethality in the larval stage [15]. The reduction of TCTP level using TCTP RNAi readily reduce the body and organ size of *Drosophila* [15]. The series of experiments reveals the conserved role of TCTP in both embryonic development and in regeneration.

The histological observation of amputation site on the fifth day of regeneration of worm injected with nutlin-3 resulted in failure in the process of wound site closure. But, quite normal wound closure was observed in the TCTP-silenced worms (**Figs 6I and 8I)**. The contrary results might be due to the following reasons. The nutlin-3 directly blocks p53 and Mdm2 association and enrich p53 level. But here, in the case of TCTP silencing, it is an indirect process of p53 transcription activation. Hence, the chances for phenotypical differences are possible. The silencing of TCTP protein expression and administration of nutlin-3 injection caused regeneration arrest (**Figs 6I and 8I)**. Similarly, it has been reported that the effect of reduction in TCTP level with nultin-3 caused mitotic arrest in human cancer cells [11] and suppression of TCTP stops cell proliferation and causes tumour reversion in cancer cells [14,61]. Interestingly, there was a very low mitotic index observed in the nutlin-3 and TCTP RNAi tissues (**Fig 12A and 12B)**. The experimental evidence highly correlates with previous reports [48]. The arrest of regeneration is not only by the inhibition of cell cycle arrest in the earthworm because the increase in longitudinal cell proliferation has been observed in the clitellar and body segments. The arrest of regeneration process should be linked with the TCTP role in signalling pathways of cell differentiation because the cells in GECL layer failed to disappear in the fifth day of regeneration in the TCTP silenced worms (**Fig 11D and 11H**). The observation clearly confirms the role of TCTP cellular changes during *E*. *eugeniae* regeneration process. The series of experiments form the milestone in functional role of TCTP in worm regeneration.

## Conclusion

The evolutionarily conserved gene, TCTP, was identified in *E*. *eugeniae* and sequenced. It has high homology with both invertebrates and vertebrates. A higher expression of TCTP was noted in early regeneration and, later, the expression was declined. The pharmacological and targeted suppression of TCTP halted regeneration. From the observations made in this experiment, we can conclude that TCTP plays a key role in the differentiation of cells in the clitellum.

## Supporting information

S1 Fig Analysis the toxicity of nutlin-3 (5 μg/g) in intact earthworm *E*. *eugeniae*. The earthworm injected with 5 μg/g concentration of nutlin-3, there is no phenotypical changes were observed.
(TIF)

S2 Fig Tunnel assay of TCTP and GFP siRNA injected tissues. (A) 40X image of GFP siRNA injected worm tissue shows no tunnel positive cells. (B) 40X image of TCTP siRNA injected worm tissue shows no tunnel positive cells.
(TIF)
