# Supplementary figures and images for: Function of translationally controlled tumor protein (TCTP) in Eudrilus eugeniae regeneration
Source: PLoS One. 2017 Apr 12;12(4):e0175319. doi: 10.1371/journal.pone.0175319 (PMC5389791; doi:10.1371/journal.pone.0175319)

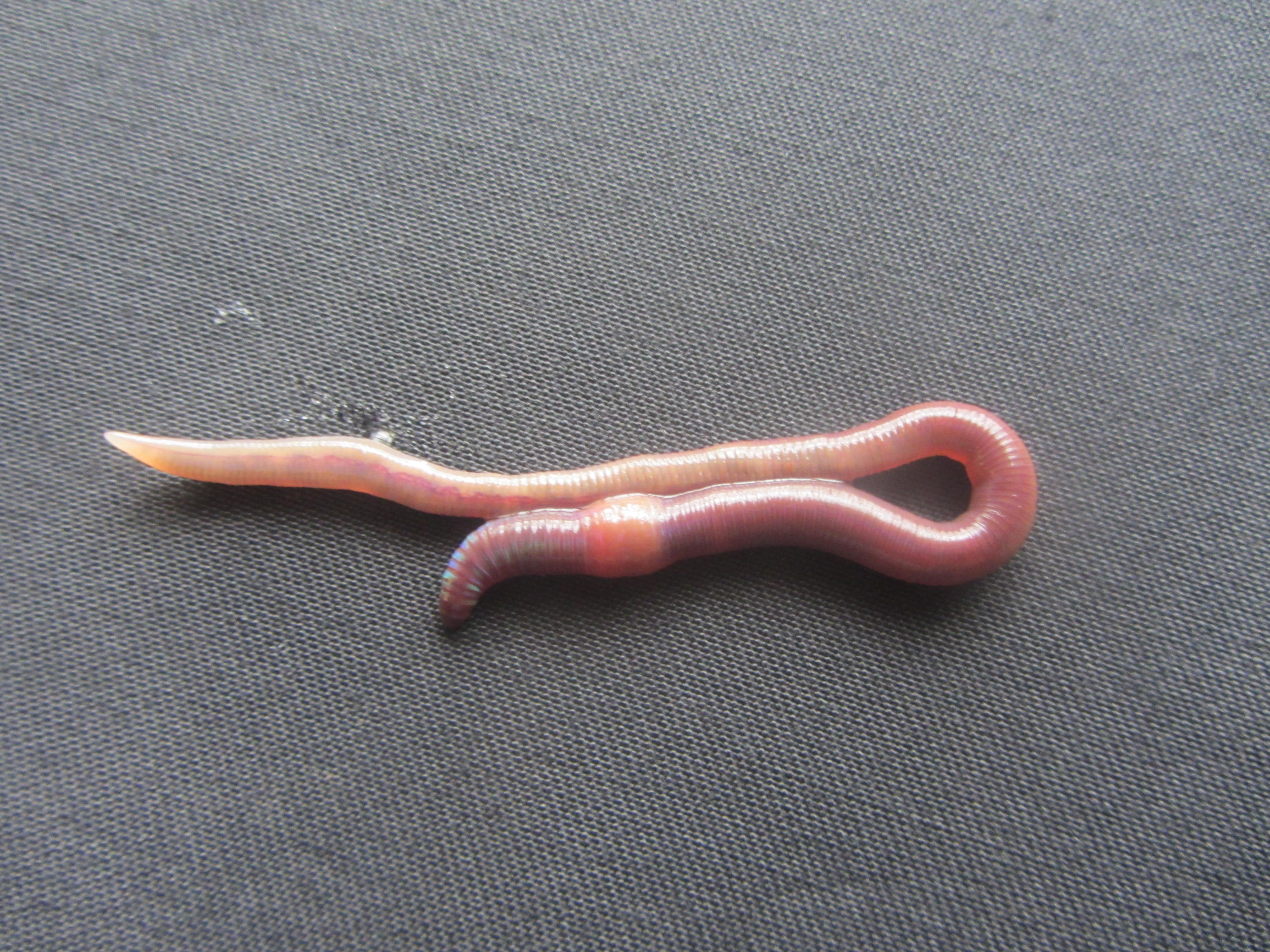

Supplement: S1 Fig — The earthworm injected with 5 μg/g concentration of nutlin-3, there is no phenotypical changes were observed. (TIF) [file pone.0175319.s001.tif]

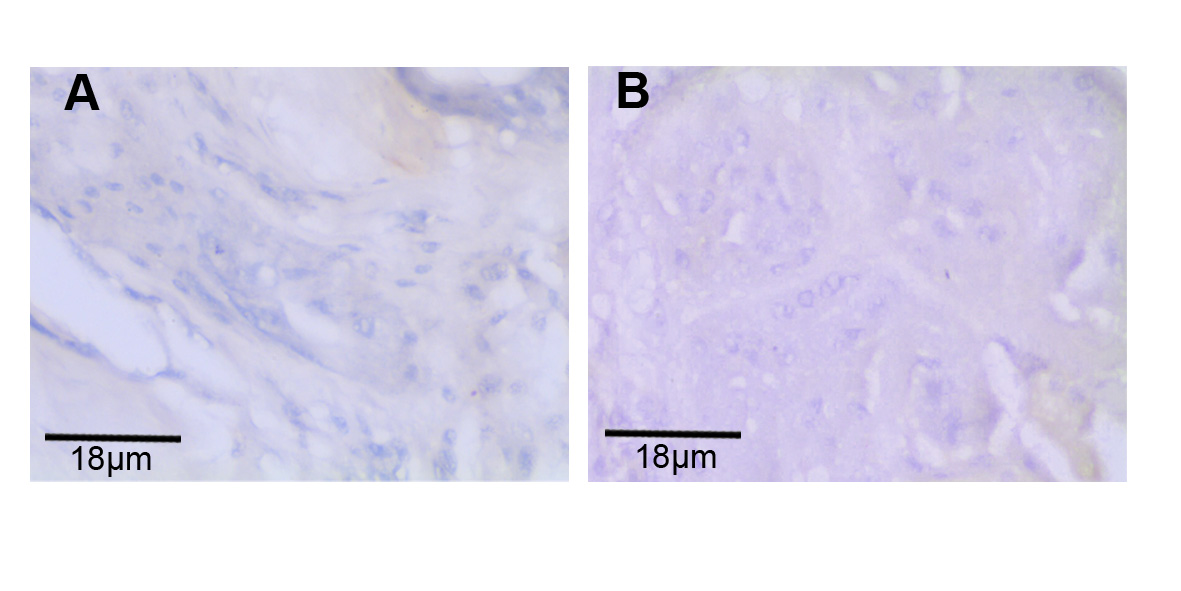

Supplement: S2 Fig — (A) 40X image of GFP siRNA injected worm tissue shows no tunnel positive cells. (B) 40X image of TCTP siRNA injected worm tissue shows no tunnel positive cells. (TIF) [file pone.0175319.s002.tif]
